# New normative standards of conditional reasoning and the dual-source model

**DOI:** 10.3389/fpsyg.2014.00316

**Published:** 2014-04-17

**Authors:** Henrik Singmann, Karl Christoph Klauer, David Over

**Affiliations:** ^1^Institut für Psychologie, Albert-Ludwigs-Universität FreiburgFreiburg, Germany; ^2^Department of Psychology, Durham UniversityDurham, UK

**Keywords:** conditional reasoning, probabilistic reasoning, new paradigm psychology of reasoning, dual-source model, coherence, p-validity, rationality, mixed models

## Abstract

There has been a major shift in research on human reasoning toward Bayesian and probabilistic approaches, which has been called a new paradigm. The new paradigm sees most everyday and scientific reasoning as taking place in a context of uncertainty, and inference is from uncertain beliefs and not from arbitrary assumptions. In this manuscript we present an empirical test of normative standards in the new paradigm using a novel probabilized conditional reasoning task. Our results indicated that for everyday conditional with at least a weak causal connection between antecedent and consequent only the conditional probability of the consequent given antecedent contributes unique variance to predicting the probability of conditional, but not the probability of the conjunction, nor the probability of the material conditional. Regarding normative accounts of reasoning, we found significant evidence that participants' responses were confidence preserving (i.e., p-valid in the sense of Adams, 1998) for MP inferences, but not for MT inferences. Additionally, only for MP inferences and to a lesser degree for DA inferences did the rate of responses inside the coherence intervals defined by mental probability logic (Pfeifer and Kleiter, 2005, 2010) exceed chance levels. In contrast to the normative accounts, the dual-source model (Klauer et al., 2010) is a descriptive model. It posits that participants integrate their background knowledge (i.e., the type of information primary to the normative approaches) and their subjective probability that a conclusion is seen as warranted based on its logical form. Model fits showed that the dual-source model, which employed participants' responses to a deductive task with abstract contents to estimate the form-based component, provided as good an account of the data as a model that solely used data from the probabilized conditional reasoning task.

## Introduction

The most influential work in the psychology of conditional reasoning long presupposed as its normative standard the binary and extensional logic of the propositional calculus (Johnson-Laird and Byrne, 1991 see especially pp. 7 and 74). In this logical system, a conditional “if *p* then *q*” is the material, truth functional conditional, which is logically equivalent to “not-*p* or *q*.” There are, however, many problems with holding that the natural language conditionals that people reason with are equivalent to material conditionals (Evans and Over, 2004). Prominent among these problems are the “paradoxes” of the material conditional. For example, it is logically valid to infer a material conditional, equivalent to “not-*p* or *q*,” from “not-*p*,” and so the probability of such a conditional will increase as the probability of “not-*p*” increases. But consider a conditional about a coin we know to be fair, “If we spin the coin 100 times then we will get 100 heads.” It would be absurd if our subjective probability for this conditional increased to ever higher levels as it became more and more likely that we would not go to the trouble of spinning the coin that many times.

Another limitation of this binary and extensional paradigm was that participants were asked in experiments on reasoning to assume that the premises were true and to give binary responses about what did, or did not, necessarily follow. In contrast, most human reasoning, in everyday affairs and science, is from uncertain premises, from more or less confidently held beliefs and statements or claims made by other people. The conclusions drawn are also more or less subjectively probable. Dissatisfaction with the traditional experiments has been a factor in the proposal of a new paradigm in the psychology of reasoning (Over, 2009; Evans, 2012; Elqayam and Over, 2013). The aim of the new paradigm is to move beyond experiments on abstract materials and premises given as assumptions. Participants are asked to reason in an everyday setting from content rich materials, and to provide their responses on graded scale reflecting various degrees of belief (see Rips, 2001; Singmann and Klauer, 2011, for an empirical dissociation of both methods).

The proposed normative system for the new studies of conditional reasoning is no longer the binary and extensional propositional calculus, but rather subjective probability theory that goes back to de Finetti (1936, 1937) and Ramsey (1931). The relevant normative standard is what de Finetti termed the logic of probability and Ramsey the logic of partial belief, as developed by Adams (1998), Gilio (2002), Gilio and Over (2012), and others. The new paradigm in the psychology of reasoning can also be seen as part of the great impact Bayesian approaches have had generally in cognitive science (Oaksford and Chater, 1994, 2001, 2007; Oaksford et al., 2000). Evans and Over (2004), Pfeifer and Kleiter (2005, 2010), and Oaksford and Chater (2007) have proposed accounts of human conditional reasoning that are central examples of the new paradigm.

Much research in the old paradigm dealt with so-called *basic* conditionals, which are defined to be indicative conditionals with an abstract content (Johnson-Laird and Byrne, 2002). People cannot use background knowledge and context to help them evaluate basic conditionals. Such conditionals are not very similar to the knowledge and context laden conditionals of ordinary and scientific reasoning. Realistic indicative conditionals, of the latter type, can be classified in a number of ways (Douven and Verbrugge, 2010), but here we are mainly concerned with conditionals that are justified by some sort of (at least weak) causal connection between the antecedent and consequent (Over et al., 2007). For lack of a better term, we call these conditionals *everyday conditionals*. Our interest in these conditionals stems from the fact that people use subjective probability judgments based on knowledge of content and context to evaluate them.

The new paradigm gives a new interpretation to such everyday conditionals. It does not see them as material conditionals, the probability of which is the same as that the probability of “¬*p* or *q*,” *P*(¬*p* ∨ *q*), (where ¬*p* is “not-*p*”). In the new paradigm, the probability of one of these conditionals, *P*(if *p* then *q*), is the conditional probability of its consequent given its antecedent, *P*(*q*|*p*). The relation, *P*(if *p* then *q*) = *P*(*q*|*p*), is so important that it is simply called the Equation (Edgington, 1995) in analytical philosophy (or *conditional probability hypothesis* in psychology)^1^. Based on the Equation probabilistic accounts of human conditional reasoning were developed (Oaksford and Chater, 2007; Pfeifer and Kleiter, 2010). Moreover, if the Equation holds, the “paradoxes” of the material conditional we referred to above cannot be derived (Pfeifer, 2014; see Pfeifer, 2013, for an empirical study of the “paradoxes”). For example, it will no longer hold that the probability of the above example conditional, about spinning the coin 100 times, increases as we become more and more determined not to spin it that many times. The conditional probability that we will get 100 heads given that we spin the coin 100 times is extremely low, and will stay low as it gets more and more likely we will not spin the coin.

Another hypothesis for the probability of everyday conditionals concerns those justified by reference to causal relations. If such conditionals state the existence of a causal relation, then the presence of the antecedent should raise the probability of the consequent compared to when the antecedent is absent. In other words, whereas the conditional probability *P*(*q*|*p*) should be positively related with the probability of a conditional, the conditional probability of alternatives to the conditional (i.e., not *p* cases leading to *q*), *P*(*q*|¬*p*) should be negatively related with the probability of a conditional. This inequality (*P*(*q*|*p*) − *P*(*q*|¬*p*) > 0) is also known as the *delta-p* rule (Allan, 1980; Sloman, 2005).

A multitude of studies has shown that the conditional probability *P*(*q*|*p*) and to a lesser extent the conjunction *P*(*p* ∧ *q*) are predictors for the probability of the conditional (Evans et al., 2003; Oberauer and Wilhelm, 2003; Oberauer et al., 2007; Over et al., 2007; Douven and Verbrugge, 2010, 2013; Politzer et al., 2010; Fugard et al., 2011). A first goal of the current manuscript is to further advance these previous studies by adopting some procedural variations which more strongly capture the central notions of the new paradigm, everyday reasoning and subjective probabilities. Specifically, some of the studies (Evans et al., 2003; Oberauer et al., 2007; Fugard et al., 2011) have, using the probabilistic truth table task, provided participants with the frequencies constituting the joint probability distribution over antecedent and consequent for a given basic conditional. From this probability distribution the probabilities corresponding to the different hypotheses for the probability of the given conditional could be construed and compared with individuals' estimates of the probability of said conditional. The probabilities used were particularly easy to grasp (see especially Politzer et al., 2010), but perceptions of probabilities can be biased (e.g., Tversky and Kahneman, 1992). In some studies the conditional probability *P*(*q*|*p*) is not reported directly by participants but calculated from their estimates of the unconditional probabilities constituting the joint probability distribution over antecedent and consequent (Over et al., 2007). As conditional probability is seen as primitive by some proponents of the new paradigm (and not defined over unconditional probabilities but given by people's use of the Ramsey test - see Evans and Over, 2004; Pfeifer and Kleiter, 2005) it may seem preferable to work with it as a primitive probability. Finally, we think it is important to show the relationship on an individual level (cf. Douven and Verbrugge, 2010, 2013). Hence, we assess the conditional probability hypothesis with everyday conditionals and assess the probabilities corresponding to the different competing hypotheses directly and independently.

In addition to the question on how individuals understand the conditional, the new paradigm also offers new ideas on how individuals reason from conditional inferences. The conditional inferences usually studied consist of the conditional as the major premise, a categorical minor premise, and a putative conclusion:

- *Modus Ponens* (MP): If *p* then *q*. *p*. Therefore *q*.
- *Modus Tollens* (MT): If *p* then *q*. Not *q*. Therefore not *p*.
- *Affirmation of the Consequent* (AC): If *p* then *q*. *q*. Therefore *p*.
- *Denial of the Antecedent* (DA): If *p* then *q*. Not *p*. Therefore not *q*.

In the next paragraphs we present major accounts for explaining reasoning from those conditional inferences within the new paradigm.

### Normative accounts

According to classical logic MP and MT are valid (i.e., truth preserving) inferences: the truth of the two premises necessarily entails the truth of the consequence. Likewise AC and DA are not valid, so the truth of the conclusion does not necessarily follow from true premises (i.e., drawing an AC or DA inference is considered a reasoning fallacy). But the new paradigm focuses generally on degrees of belief in premises. A normative view that builds upon degrees of belief is given by Adams' (1998) probability logic with the notion of probabilistic validity or p-validity, according to which inferences should be *confidence preserving*: a p-valid conclusion cannot be more *uncertain* than the premises on which it is based. Formally, uncertainty of an event *p* is defined as the complement of the probability of *p*, *U*(*p*) = 1 − *P*(*p*), and for p-valid inferences the uncertainty of the conclusion cannot exceed the sum of the uncertainties of the premises whatever the probabilities of the premises and conclusion. Parallel to classical logic, MP and MT are p-valid and AC and DA are not p-valid. Hence another goal of the current manuscript is to provide a test of p-validity as a computational level account (in Marr's, 1982, sense) of human reasoning.

A stronger normative framework is proposed by Pfeifer and Kleiter's (2005; 2010) *mental probability logic*, as it derives probabilistically informative restrictions for all four inferences, MP, MT, AC, and DA. In contrast to Adams' (1998) notion that valid inferences are confidence preserving, they propose that reasoners' inferences should be probabilistically *coherent* (see de de Finetti, 1936; Coletti and Scozzafava, 2002; Gilio, 2002). Coherence here means that reasoners, when asked to estimate the probability of an event that stands in a relationship with other events for which the probabilities are known or estimated (e.g., the conclusion derived from a set of premises), should make an estimate that does not expose them to a Dutch book (i.e., that is coherent with the other probabilities according to coherence-based probability logic; see Pfeifer, 2014, for the relation to standard probability theory). Furthermore, in case not all probabilities necessary to calculate a point estimate for the desired event are available, the estimated probability should fall in the interval that is derived when the missing probabilities are allowed to range between 0 and 1. For example, in the case of MP, the two premises are (a) the conditional statement *if p then q* with probability *P*(*q*|*p*) and (b) the minor premise *p* with probability *P*(*p*) and the to be estimated probability is *P*(*q*), for the conclusion *q*. According to the law of total probability the desired probability is given by:

(1) MP: P(q)=P(q|p)P(p)+P(q|¬p)(1−P(p))

Note that we have exchanged the probability *P*(¬*p*) with its complement 1 − *P*(*p*) with the consequence that of the four terms on the right side, three are already present in the premises. The product of the premises is the first summand, *P*(*q*|*p*)*P*(*p*), and the complement of the minor premise, 1 − *P*(*p*) is present in the second summand. Only the probability of alternatives to the conditional, *P*(*q*|¬*p*) (i.e., non-*p* cases in which the consequent holds), is less salient given that none of the premises concerns this probability. Assuming that *P*(*q*|¬*p*) can range from 0 to 1, we can substitute it with either 0 or 1 which gives us the coherence interval for MP:

MP: P(q)=[P(q|p)P(p),P(q|p)P(p)+(1−P(p))].

Pfeifer and Kleiter (2005; see also Wagner, 2004; Pfeifer and Kleiter, 2006) provide analogous intervals for the other inferences:

$$\begin{array}{l} \text{MT: }P(\neg p)=[\text{max}(\frac{1-P(q|p)-P(\neg q)}{1-P(q|p)},\\ \;\frac{P(q|p)+P(\neg q)-1}{P(q|p)})\text{​},1] \end{array}$$

$$\text{AC: }P(p)=\left[0,\text{min}\left(\frac{P(q)}{P(q|p)},\frac{1-P(q)}{1-P(q|p)}\right)\right]$$

$$\begin{array}{l} \text{DA: }P(\neg q)=[1-P(\neg p)-P(q|p)(1-P(\neg p)),\\ \;1-P(q|p)(1-P(\neg p))] \end{array}$$

One goal of the current manuscript is to test mental probability logic as a computational levels account of reasoning, by assessing whether or not participants responses fall in the intervals predicted by mental probability logic.

Another normative account of conditional reasoning stems from the proponents of Bayesian rationality, Oaksford and Chater (2007, chapter 5; Oaksford et al., 2000). Their probabilistic approach is couched within the same philosophical tradition as the aforementioned ones and also uses elementary probability theory to derive predictions but differs in one important aspect. It assumes that the presence of the minor premise sets the corresponding probability to one [e.g., *P*(*p*) = 1 for MP]. The assumed inferential step to derive an estimate of the conclusion is to conditionalize on the minor premise, the probability of the conclusion should equal the conditional probability of the conclusion given minor premise. For example for MP, the probability of the conclusion should equal the probability of the conditional, *P*(*q*|*p*). Oaksford and Chater provided formulas to obtain point estimates for all four inferences. However, in the study reported in this manuscript we employed an experimental method in which the subjective probability of the minor premise need not equal 1. Therefore, we do not test the empirical adequacy of Oaksford and Chater's account as a computational level theory of reasoning. We follow Pfeifer and Kleiter (2006; 2007; 2009; 2010) and Evans et al. (2014) in studying whether people conform to p-validity and coherence in their conditional inferences when both premises are uncertain.

### The dual-source model

A formal model for a descriptive account for probabilistic reasoning was proposed by Klauer et al. (2010), the *dual-source model*. It assumes that individuals integrate two different types of information (i.e., sources) when making an inference, background knowledge regarding the subject matter and information regarding the logical form of the inference. The background knowledge reflects individuals' subjective probability with which the conclusion follows from the premises given the individuals' knowledge about them. This part of the model is tied to the normative approaches presented so far in that the model assumes that for conditional inferences this probability is derived from a coherent probability distribution over *p*, *q*, and their complements. In fact, the published studies employing the dual-source model used the formulas by Oaksford et al. (2000) to estimate the knowledge based component.

The theoretical expansion to the probabilistic approaches presented so far is the form-based component. It reflects individuals subjective probability with which an inference is warranted by the logical form (e.g., “How likely is the conclusion given that the inference is MP?”). The introduction of this part of the model was in part motivated by empirical findings that participants give higher estimate to a conclusion *q* when in addition to the minor premise *p* the conditional “if *p* then *q*” is also present (Liu, 2003). In other words, only conditionalizing on the minor premise, as proposed by Oaksford et al. (2000), does not seem to capture the complete data pattern. It should be noted that the form-based component is a subjective probability reflecting participants' belief in the logicality of logical forms and thereby not directly related to the actual logical status. To come to a blended reasoning conclusion the knowledge-based information, represented by parameter ξ, and the form-based information, represented by parameter τ, are integrated by the weighting parameter λ using Bayesian model averaging. The prediction of the dual-source model for a conditional *C* and inference *x* is given by

(2) λ{τ(x)+(1−τ(x))×ξ(C,x)}+(1−λ)ξ(C,x)

Note that in this formula, the knowledge parameters ξ(*C*, *x*) enters the model in two places: in the knowledge-based component [the second summand which is weigthed with (1 − λ)], but also in the form-based part (weighted with λ). The rationale for the latter is that it is assumed that individuals, in cases when they are unsure of whether or not a conclusion is warranted by the logical form of the inference (i.e., in (1 − τ(*x*)) cases), resort to their background knowledge, ξ(*C*, *x*), as a fall-back position. One goal of the present manuscript is to apply the dual-source model in an experimental setup that strongly diverges from the experiments reported by Klauer et al. (2010), thereby providing convergent evidence for its usefulness. Furthermore, we use (a) a different formula to estimate the knowledge-based component which is based on the ideas of mental probability theory and uses no free parameters and (b) use a novel way to estimate the form-based component of the model which also does not rely on free parameters.

## The present experiment

For an empirical test of the empirical adequacy of the approaches presented above it is necessary to obtain not only participants' responses to the conditional inferences, but also estimates of the probability of the premises and estimates of the hypothesized predictors for the probability of the conditional. Therefore, participants provided the probabilities necessary to test the aforementioned approaches in addition to estimating the probability of the conditional inferences. In this novel *probabilized conditional inference task*, participants were first asked for the probability of the conditional and then for the probability of the minor premise. Next we presented the conditional inference: the conditional and minor premise were presented together with participants' probability estimates for the premises. Participants were asked to estimate how likely the conclusion is given the information presented. After this, we asked for the remaining probabilities we were interested in for this specific content, such as the conditional probability *P*(*q*|*p*) or the probability of alternatives to the conditional, *P*(*q*| ¬*p*). To use this order invariantly, participants only worked on one inference for each conditional. In line with our goal to assess everyday reasoning we only used highly believable conditionals, as reasoning from unbelievable conditionals seems somewhat unnatural. To obtain estimates of participants' form-based components of the dual-source model participants performed a second task afterwards. They worked on a *deductive conditional inference task* with abstract materials and strong deductive instructions (see Singmann and Klauer, 2011).

## Methods

### Participants

Thirty participants (mean age = 22.4 years, *SD* = 2.9, range from 18 to 30 years) participated in this experiment which was the second session in a larger study on reasoning addressing other hypotheses with other materials. In the previous session participants had worked on a conditional inference task with probabilistic instructions. More specifically, in the previous session participants were asked to provide estimates for the probability of the conclusions of all four conditional inferences (plus for the four so-called *converse inferences*; Oaksford et al., 2000) for six different conditionals three of which were uttered by an expert and three by a non–expert (i.e., analogous to Stevenson and Over, 2001). Sessions were separated by at least 1 week. Most participants were students of the University of Freiburg (28) with differing majors, excluding majors with an education in logic such as math, physics or psychology. Participants received 14€ compensation after the third session.

### Materials

All materials were presented in German, participants' mother tongue. For the probabilized conditional reasoning task we adapted 13 believable conditionals from Evans et al. (2010) and added three similar conditionals (which were not pretested) such as “If Greece leaves the Euro then Italy will too.” The full list of conditionals can be found in the Supplemental material. Each participant worked on four randomly selected conditionals of the total of 16 conditionals and performed only one inference (i.e., MP, MT, AC, or DA) per conditional. More details are given below. In the instructions it was clarified that the conditionals were related to events that might occur within the next ten years in Germany or the rest of the world.

For the deductive conditional inference task we used two conditionals about a hypothetical letter number pair: “If the letter is a B then the number is a 7.” and “If the number is a 4 then the letter is an E”. Participants performed all four inferences for both conditionals.

### Procedure

#### Probabilized conditional inference task

In the first part of the experiment, participants were instructed to estimate probabilities of events or statements or to estimate the probability of a conclusion following an argument, “as if they were in a discussion regarding these issues.” Four conditionals were randomly selected for each participant and randomly assigned to the four inferences. For each conditional/inference participants responded to eight items which were presented in one block (i.e., participant first responded to all eight items for the conditional that was randomly selected for e.g., MP, before working on the eight items for the conditional that was randomly selected for e.g., MT). As participants only worked on exactly one conditional for each inference, participants worked on four blocks of eight items in total (i.e., 32 items overall in the probabilized conditional inference task) and the order of blocks was also randomized anew for each participant. For each item, the response was given on a scale from 0 to 100%. In contrast to the work of Pfeifer and Kleiter (e.g., 2007, 2010), who asked participants to provide either point estimates or interval estimates, participants in our experiments always had to provide point estimates, even for the conditional inference [type (b) below]. The responses were transformed to a probability scale (i.e., divided by 100) prior to the analysis. Each item appeared on its own screen.

Within each block, participants responded to three different types of items: (a) first participants gave estimates for the *probability of the premises*, (b) then participants had to estimate the *conclusion of the conditional inference*, and (c) finally participants had to estimate the *other probabilities* we were interested in. The three different types of items were always presented in that order. In the following we present one example for each of the eight items using the conditional “If Greece leaves the Euro then Italy will too”, assuming it was randomly selected for the MP inference. For items of type (a) (probability of the premises), participants first estimated the probability of the conditional, *P*(if *p* then *q*), and then the probability of the minor premise, *P*(*p*).

If Greece leaves the Euro then Italy will too.
In your opinion, how probable is the above statement/assertion [Aussage]?
Greece will leave the Euro.
In your opinion, how probable is it that the above event occurs [dass die obige Aussage eintritt]?

There was only one item of type (b): Participants had to give an estimate of the probability of the conclusion following the conditional inference. They, were again presented the conditional and the minor premise along with the probability estimates participants had just given (represented by *xx*):

If Greece leaves the Euro then Italy will too.
(Probability *xx*%)
Greece will leave the Euro.
(Probability *xx*%)
Under these premises, how probable is that Italy will leave the Euro, too?

After this, the items of type (c) for the other probabilities we were interested in were presented in a new random order for each block and participant. For evaluating the conditional probability hypothesis, we asked participants to estimate the conditional probability *P*(*q*|*p*), the probability of the conjunction *P*(*p* ∧ *q*), and the probability of the material conditional *P*(¬*p* ∨ *q*). Furthermore we asked for the probability of alternatives, *P*(*q*|¬*p*), and again for the probability of the event in the conclusion this time without the premises (i.e., *P*(*q*) for MP, *P*(¬*p*) for MT, *P*(*p*) for AC, and *P*(¬*q*) for DA; however, we do not report an analysis of this estimate in the following):

*P*(*q*|*p*):
How probable is that Italy will leave the Euro should Greece leave the Euro?
*P*(*p* ∧ *q*):
Greece will leave the Euro and simultaneously Italy will leave the Euro.
In your opinion, how probable is it that the above event occurs?
*P*(¬*p* ∨ *q*):
Greece will NOT leave the Euro or Italy will leave the Euro.
In your opinion, how probable is it that the above event occurs?
*P*(*q*|¬*p*):
How probable is that Italy will leave the Euro should Greece NOT leave the Euro?
*P*(*q*):
Italy will leave the Euro.
In your opinion, how probable is it that the above event occurs?

After working on all eight items for one combination of inference and conditional, participants then worked on the next block of eight items with a different combination of inference and conditional. Note that in blocks for inferences other than MP, the questions for the minor premise [type (a)] and the question for the conclusion [type (b) and type (c), last item] were adapted accordingly (but no other questions).

#### Deductive conditional inference task

Directly after the first task the second task started, which was modeled after Singmann and Klauer's (2011) deductive condition. Participants were instructed to judge the logical validity of arguments: “Which conclusion follows with logical necessity from a given argument?” The response had to be given on a scale from 0 to 100 (i.e., the same scale as in the probabilized task, but without the %-character). For example:

If the number is a 4 then the letter is an E.
The number is a 4.
How valid is the conclusion that the letter is an E from a logical perspective?

Participants were instructed to respond with 0 if the conclusion did not necessarily follow from the premises and with 100 if the conclusion did necessarily follow from the premises. Furthermore they read: “When you are unsure, you can indicate the degree to which you think the conclusion is valid by selecting a number between 0 and 100.” Participants worked on all four inferences for each of the two conditionals. Presentations of inferences was random, blocked per conditionals, with the blocks also presented in random order. The responses were transformed to a probability scale (i.e., divided by 100) prior to the analysis.

## Results and discussion

### Conditional probability hypothesis

The conditional probability hypothesis states that the probability of the conditional *P*(if *p* then *q*) is predicted by the conditional probability *P*(*q*|*p*), whereas neither the probability of the material conditional *P*(¬*p* ∨ *q*) nor the probability of the conjunction *P*(*p* ∧ *q*) should contribute unique variance to this prediction. According to the delta-p rule, the probability of the conditional should also be negatively related to the probability of alternatives *P*(*q*|¬*p*). Table 1 displays the correlations of these variables across all responses (i.e., item by participant combinations). It can be seen that, as predicted, the conditional probability *P*(*q*|*p*) and additionally the conjunction *P*(*p* ∧ *q*) are correlated with *P*(if *p* then *q*) but not the other variables. However, these results have to be interpreted cautiously as responses were nested within participants (each participant gave four responses) and within conditionals (for each conditional we obtained between five and ten responses) which violates the assumptions for standard correlation or multiple regression (Judd et al., 2012).

**Table 1:** **Correlations with the Probability of the Conditional *P*(if *p* then *q*)**.

	***P*(*q*|*p*)**	***P*(*p* ∧ *q*)**	***P*(¬*p* ∨ *q*)**	***P*(*q*|¬*p*)**	**Mean**	***SD***
*P*(if *p* then *q*)	0.84^*^	0.61^*^	0.09	0.04	0.61	0.26
*P*(*q*|*p*)		0.72^*^	0.11	0.08	0.60	0.27
*P*(*p* ∧ *q*)			0.15	**0.21**	0.54	0.30
*P*(¬*p* ∨ *q*)				0.43^*^	0.42	0.25
*P*(*q*|¬*p*)					0.27	0.24

Significant correlations (p < 0.05) are printed in bold. Correlations that are also significant after controlling for multiple testing using the Bonferroni-Holm correction are additionally marked with an asterisk. The two rightmost columns show mean and SD of the variables.

To overcome these problems, we estimated a linear mixed model (LMM) for the probability of the conditional as dependent variable with crossed random effects for participants and conditional (Baayen et al., 2008) using lme4 (Bates et al., 2013) for the statistical programming language R (R Core Team, 2013). We entered the four assumed predictors and inference (MP, MT, AC, and DA) simultaneously as fixed effects and estimated random intercepts for participants and items plus random inference slopes and correlations among the random inference slopes for the random item effect. This model realized the *maximal random effects structure* recommended by Barr et al. (Barr, 2013; Barr et al., 2013), the random inference slopes for participants had only one observation for every level and could therefore not be estimated reliably)^2^. A model without the fixed and random effects for inference produced the exact same pattern of significant and non–significant results. To assess the significance of fixed effects in LMMs we obtained the Kenward-Rogers approximation for degrees of freedom of the full model compared with a model in which the effect of interest was excluded throughout this manuscript with the methods implemented in afex (Singmann, 2013) and pbkrtest (Halekoh and Højsgaard, 2013). The fixed effects are displayed in Table 2 and were fully in line with the conditional probability hypothesis: when controlling for participant and item effects and estimating all parameters simultaneously, only the conditional probability *P*(*q*|*p*) was a significant predictor of the probability of the conditional and none of the other variables. In fact, for all other predictors the estimated parameters were virtually 0.

**Table 2:** **Main effects linear mixed model on the probability of the conditional *P*(if *p* then *q*)**.

**Effect**	**Parameter**	***F***	***df***	**F-scaling**	***p***
(Intercept)	0.14	8.70	1, 60.35	1	0.005
Inference		0.43	3, 10.16	0.84	0.74
*P*(*q*|*p*)	0.78	86.81	1, 88.14	1	<0.001
*P*(*p* ∧ *q*)	0.00	0.00	1, 90.91	1	>0.99
*P*(¬*p* ∨ *q*)	−0.01	0.01	1, 88.26	1	0.91
*P*(*q*|¬*p*)	−0.00	0.00	1, 81.59	1	0.98

The model was fitted with restricted maximum likelihood. Model df = 20, AIC = −98.42, BIC = −42.67, deviance = −138.42, Ω^2^_0_ = 0.84 (explained variance against the intercept only model; Xu, 2003). The values in the table note are based on a model with variables centered on 0 to be comparable to the Supplemental material.

**Figure 1:**
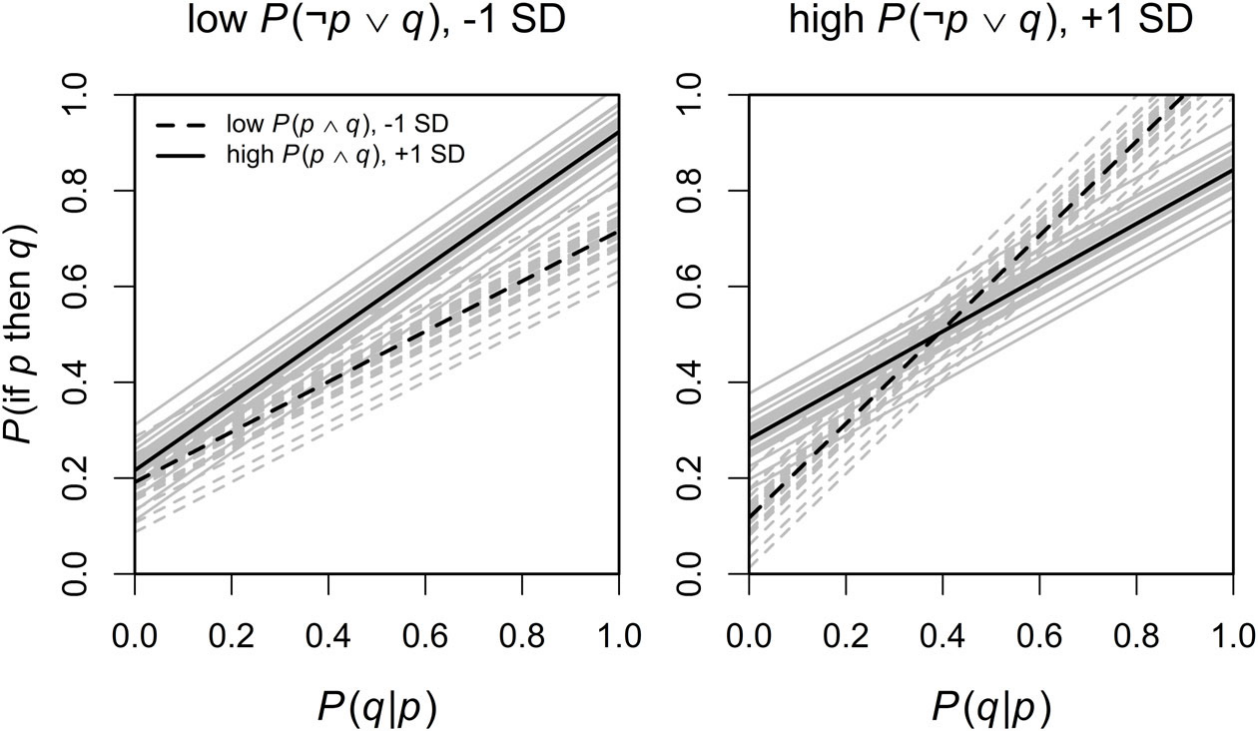
**The significant three-way interaction of *P*(*q*|*p*) × *P*(*p* ∧ *q*) × *P*(¬*p* ∨ *q*) on *P*(if *p* then *q*)**. Predictions based on the random participant effects are shown in gray, and the mean effect in black. Detailed description in the main text.

In an exploratory analysis we estimated a second mixed model in which we added all interactions of the predictors of interest (after centering all predictors and the dependent variable on 0). The random effects structure remained identical to the previous model. In an additional exploratory analysis in which we excluded the random and fixed effects for inference, the pattern of significant and non–significant effects was the same as reported below. The analysis revealed, in addition to the significant main effect of *P*(*q*|*p*), a significant three-way interaction of *P*(*q*|*p*) with *P*(*p* ∧ *q*) and *P*(¬*p* ∨ *q*), *F*_(1, 72.74)_ = 4.09, *p* = 0.047 (the full results table can be found in the Supplemental material). This interaction is displayed in Figure 1, with the main predictor *P*(*q*|*p*) on the *x*-axis and the dependent variable *P*(if *p* then *q*) on the *y*-axis, high and low values of *P*(*p* ∧ *q*) are displayed as separate lines and high and low values of *P*(¬*p* ∨ *q*) are displayed as separate plots (with high and low values referring to values plus and minus one SD from the mean, Cohen et al., 2002). The mean values are displayed as black lines and the individual estimates based on the random participant intercepts are displayed as gray lines in the background. Predictions were obtained by setting *P*(*q*|¬*p*) to 0, aggregating across all four inferences, and then transforming the predictions back on the probability scale. This interaction indicated that for low values of *P*(¬*p* ∨ *q*), higher values for *P*(*p* ∧ *q*) also meant higher values for *P*(if *p* then *q*), whereas for high values of *P*(¬*p* ∨ *q*), *P*(*p* ∧ *q*) interacted with *P*(*q*|*p*) so that for high values of *P*(*q*|*p*) lower values of *P*(*p* ∧ *q*) predicted higher *P*(if *p* then *q*).

In summary, our data corroborated the conditional probability hypothesis: in contrast to previous work (e.g., Over et al., 2007; Fugard et al., 2011) only the conditional probability *P*(*q*|*p*) is a significant predictor of the probability of the conditional. There was no evidence in support of the other hypotheses. Although we found an unexpected three-way interaction involving *P*(*p* ∧ *q*) and *P*(¬*p* ∨ *q*), the interaction is not easy to interpret and without proper replication we refrain from discussing it further. Our second mixed model analysis revealed another interesting finding: there does not seem to be any influence of alternatives to the conditional *P*(*q*|¬*p*). If the delta-p rule influenced the subjective probability of a conditional we would expect to find either a main effect or an interaction of *P*(*q*|¬*p*) with *P*(*q*|*p*). As neither of those appeared delta-p is not supported by our data.

### p-validity

**Figure 2:**
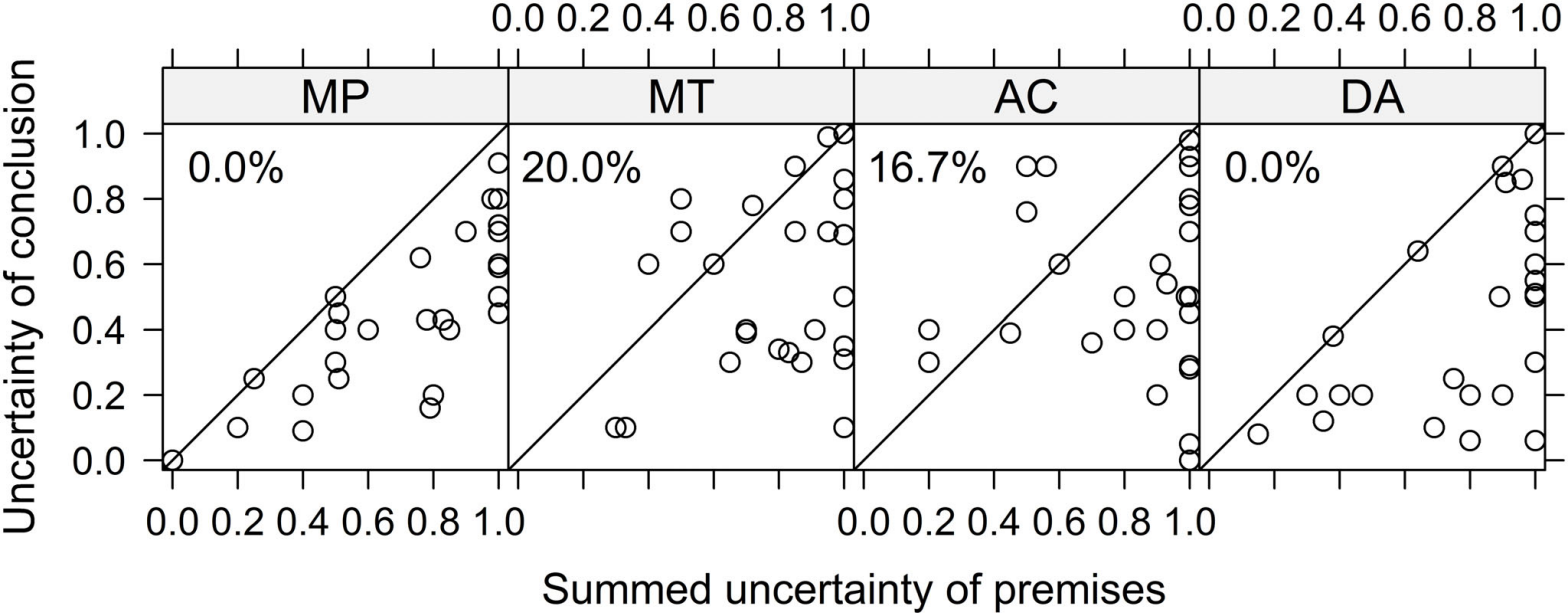
**Uncertainty of conclusion (x-axis) vs. summed uncertainties of premises (y-axis) for the four conditional inferences**. Each data point shows a single response to one inference. For MP and MT, *p*-validity is violated if data points fall in the upper triangle (i.e., uncertainty of the conclusion is larger than the uncertainty of the premises). For AC and DA no such restriction exists. The numbers in the upper left corner show the percentages of data points in the upper triangle.

According to Adams (1998), the p-valid inferences MP and MT are confidence preserving: the uncertainty of the conclusion should not exceed the summed uncertainty of the premises, where uncertainty is defined as *U*(*p*) = 1− *P*(*p*). Figure 2 displays the summed uncertainties of the premises on the x-axis against the uncertainty of the conclusion on the y-axis, for the individual responses to the four inferences (summed uncertainties larger than 1 are truncated at 1). Values in the lower triangle of each panel are consistent with p-validity and values in the upper triangle can be considered violations of p-validity (this only refers to MP and MT as there is no restriction for AC and DA). The numbers in the upper left corner of each plot are the percentage of data points in the upper triangle (i.e., violations for MP and MT). Inspection of the figure reveals that there are no violations of p-validity for the forward inference MP, but there are 20% violations for the backward inference MT. One interesting finding emerges when looking at the two inferences that are not restricted by p-validity: They mimic the pattern found for MP and MT. For the other forward inference, DA, there are also no responses in the upper triangle, whereas for the other backward inference, AC, 17% of the responses are also in the upper triangle.

The analysis so far did not take into account that the larger the summed uncertainty of the premises, the larger the probability that the response to the conditional inference is p-valid (i.e., in the lower triangle) just by chance. In the extreme case of summed uncertainties of 1 (e.g., if the probabilities of the premises are.5 each or lower) the probability of giving a p-valid response is also 1. In this case, participants cannot give a response that is not p-valid, because every possible response is. When assuming that for a chance response any value is equally likely (i.e., responses are uniformly distributed across the response scale), one can control for this chance factor in the following way, as suggested by Jonathan Evans and colleagues (Evans et al., 2014). We computed a binary variable of whether or not a given response is p-valid (coded with 1) or not (coded with 0) and compared it with the sum of the uncertainties of the premises (truncated at 1), as this gives the probability of giving a p-valid response by chance. If the difference of these two variables would be above 0, the rate of responses being p-valid would be larger than expected by uniformly distributed random responses and thus it would constitute evidence for above chance p-valid responses.

Therefore we estimated a LMM with this difference score as dependent variable with inference (MP vs. MT) as fixed effects and random intercepts for participants plus random intercepts and random inference slopes for items. The analysis showed that overall the intercept was significant, *F*_(1, 10.48)_ = 8.39, *p* = .02, indicating that there was evidence for above chance performance. However, the effect of inference was also significant, *F*_(1, 28.98)_ = 8.41, *p* = 0.007, indicating that the inferences differed in their degree of over chance performance. In fact, *post-hoc* analysis using the methods implemented in multcomp (Bretz et al., 2011) revealed that only for MP was the estimated effect of 0.26 reliably above zero, *z* = 4.21, *p* < 0.001. In contrast, for MT, the effect was estimated to be virtually 0 (−0.004) and consequently not significant, *z* = −0.06, *p* = 0.52. In this *post-hoc* analysis we used directional (i.e., one sided) hypotheses and the Bonferroni-Holm correction to control for alpha error cumulation.^3^ As some of the violations of p-validity seemed to be rather mild violations (i.e., relatively near to the diagonal of Figure 2), we repeated the reported analysis after adding 0.05 and then again after adding another 0.05 (i.e.,.1 in total) to the summed uncertainty of the conclusion to take minor deviations into account. These two alternative analyses yielded the exact same pattern of significant and non–significant results.

Taken together, this analysis shows that for MP, participants give p-valid inferences. In contrast, for MT individuals do not strictly draw p-valid conclusions, but sometimes are more uncertain about the conclusions than implied by the premises. Although some of those violations appear to be only mild violations (i.e., the problematic data points are near the diagonal) the analysis that takes chance into account indicates that there is overall no evidence for p-validity above chance for MT. This difference between MP and MT resembles the well-known asymmetry found in conditional reasoning with deductive instructions that individuals are more likely to endorse MP than MT inferences (e.g., Schroyens and Schaeken, 2000, Figure 4).

### Coherence

**Figure 3:**
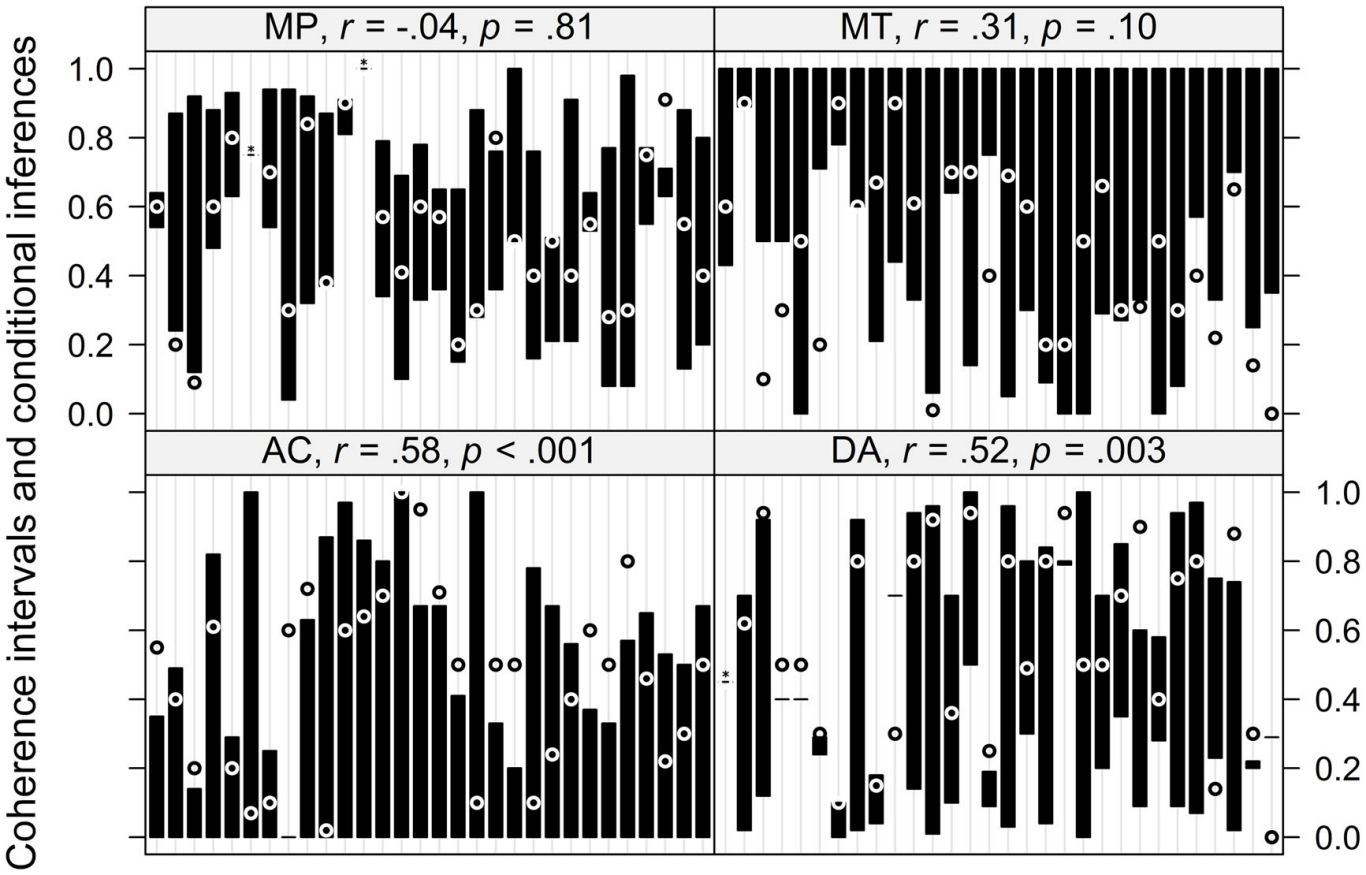
**Individual coherence intervals as predicted by mental probability logic and corresponding responses to the conditional inferences**. The intervals are depicted by black bars, responses inside the interval are depicted as a white “◦,” responses outside the interval are depicted as a black “◦.” Three cases in which the interval was only of length 0.01 but the response inside the interval are marked with an asterisk. The correlation depicted in the header of each panel is the correlation of the size of each interval with whether or not a response falls within the interval. Within each panel, the x-axis is ordered by participant ID.

In the next analysis we calculated coherence intervals based on mental probability logic (Pfeifer and Kleiter, 2005, 2010) for each individual response using the probability estimates of the premises. The intervals and the corresponding responses given to the conditional inferences are displayed in Figure 3. From this figure it is apparent that not all responses are coherent (i.e., fall within the coherence interval), however, some of those violations are very near the interval borders. Similar to p-validity, responses can fall within the intervals predicted by mental probability logic simply by chance (i.e., the larger the interval, the larger the chance to give a response within the interval). Therefore, we first looked at the correlations of the size of the interval with whether or not a response is coherent, which are given in the header of each panel in Figure 3. For MP there is clearly no such relationship. There is slight evidence for this correlation for MT and a clear correlation for both AC and DA.

Next, we performed an analysis similar to the one reported for p-validity (again following Evans et al., 2014). For each participant and response we calculated whether or not a response falls within the interval or not (coded as 1 or 0, respectively) and compared it with the size of the interval as the chance level to give a response within the interval (again assuming that random responses are uniformly distributed across the response scale). These values (as percentages) are given in Table 3, which also contains those percentages for intervals that are extended by 0.05 or 0.1 beyond the coherence intervals. To assess if observed rates of coherent responses were larger than the chance rate of coherent responses, we estimated a LMM with the difference between both variables as dependent variable with inference (MP, MT, AC, vs. DA) as fixed effect and random intercepts for participants plus random intercepts and random inference slopes for items. The analysis revealed a significant intercept, *F*_(1, 16.07)_ = 7.37, *p* = 0.02, indicating above chance performance, and a marginally significant effect of inference, *F*_(3, 9.26)_ = 2.88, *p* = 0.09. A *post-hoc* analysis analogous to the one reported above revealed that only MP showed a significant above chance performance of 0.40, *z* = 4.14, *p* < 0.001. The only other effect that was not estimated to be virtually 0 was DA with 0.14. However, this effect did not reach significance, *z* = 1.61, *p* = 0.16 (this effect almost reached significance, *p* = 0.053, when not controlling for alpha error cumulation). The effects for MT and AC (−0.02 and 0.02, respectively), did not differ from zero, *z* = −0.21, *p* = 0.82 and *z* = 0.22, *p* = 0.82, respectively. When repeating this analysis with the extended intervals the pattern of significant and non–significant results stayed basically the same, with the only exception that for the extended intervals, the *p*-values for the effect of DA dropped below 0.05 even when controlling for alpha error cumulation.

**Table 3:** **Percentage of coherent responses/coherent responses predicted by chance**.

**interval**	**MP**	**MT**	**AC**	**DA**
+/− 0	87%/45%	63%/65%	60%/58%	60%/46%
	(7%, 0.03; 7%, 0.12)	(37%, 0.17)	(40%, 0.18)	(10%, 0.29; 30%, 0.10)
+/− 0.05	97%/54%	73%/69%	63%/62%	67%/54%
	(0%, 0; 3%, 0.15)	(27%, 0.22)	(37%, 0.15)	(10%, 0.24; 23%, 0.05)
+/− 0.1	97%/63%	73%/73%	73%/67%	83%/61%
	(0%, 0; 3%, 0.10)	(27%, 0.18)	(27%, 0.12)	(7%, 0.24; 10%, 0.04)

Percentages of responses within the coherence intervals/percentages of responses within the coherence intervals predicted from the size of the intervals. The numbers in parentheses below each row are the percentages of responses below and above the interval (only below for MT and only above for AC) and the median distance of the outside responses from the border of the interval. Rows 2, 3 present the same information but with intervals extended on both sides by 0.05 and 0.1, respectively.

Our analysis of the predictions of mental probability logic reveals that, similar to p-validity, participants do not strictly adhere to coherence. In fact, only for MP and to a lesser degree for DA do we find above chance performance. In addition, it should be noted that Table 3 shows that the distance of incoherent responses from the border of the intervals is relatively large, at least for MT and AC, indicating that these outside responses are clear violations.

### The dual-source model

#### Deductive conditional inference task

**Figure 4:**
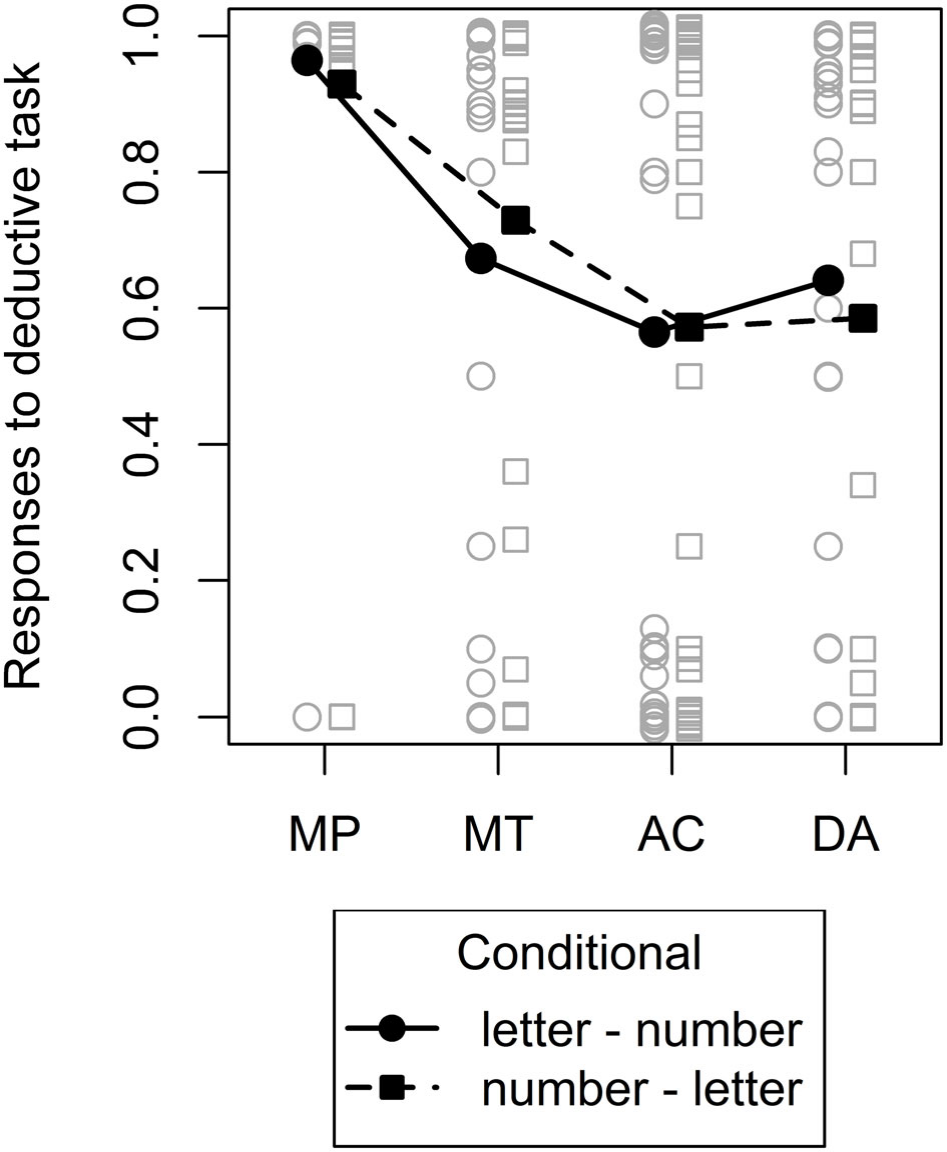
**Mean (filled symbols) and individual (non–filled symbols) responses to the deductive conditional inference task**. The two different conditionals are depicted by different lines and symbols. A small amount of vertical jitter was added to individual responses in case of perfect overlap to make points distinguishable.

To fit the dual-source model to the data we combined estimates from the probabilized conditional inference task which provided the basis for the knowledge-based component of the dual-source model (more below) with the deductive conditional inference task which provided estimates for the form-based component of the dual-source model. In the latter task, we expected participants to display a pattern of results that would be consistent with what is usually found in experiments with deductive instructions and basic conditionals (e.g., Evans, 1993): Almost unanimous endorsement of MP, lower endorsement of MT, and still lower endorsement of AC and DA, with the latter two not necessarily differing. This expected pattern is essentially what we found, as evident from Figure 4 and an LMM on the responses with inference and conditional and their interaction as fixed effects and random intercepts for participant plus random slopes for inference and conditional. We only found a significant effect of inference, *F*_(3, 27)_ = 9.58, *p* < 0.001, other *F* < 1. Planned comparisons using multcomp (Bretz et al., 2011) with directional hypotheses and no alpha-error correction revealed that indeed, endorsement for MP was higher than for MT, *z* = 2.87, *p* = 0.002, and endorsement for MT tended to be higher than endorsement for AC and DA, *z* = 1.36, *p* = 0.09, whereas there were no differences between AC and DA, *z* = −0.38, *p* = 0.65.

#### Specifying the model(s)

As already mentioned in the introduction, our method to estimate the dual-source model diverged from the parametrization used by Klauer et al. (2010). In particular, similar to Klauer et al. we assumed that participants' estimates of the probability of the conclusion from their background knowledge should follow from a coherent joint probability distribution over *p*, *q* and their complements. But in contrast to the original formalization which was based on Oaksford et al. (2000), we here follow the formalization of mental probability logic (Pfeifer and Kleiter, 2005, 2010) in that we assume that the law of total probability (as expressed in Equation 1 for MP) is the appropriate formula to describe this component (the corresponding formulas for the other inferences can be construed by elementary algebra^4^). Note that Klauer et al.'s task and the presented probabilized conditional inference task differ in that the minor premise was presented as certain in the former case and as uncertain here. The difference to the coherence intervals proposed by Pfeifer and Kleiter is that we use participants' estimate of the probability of alternatives to the conditional, *P*(*q*|¬*p*), which they provided after making the conditional inference, to obtain point estimates of the knowledge-based component.

The *baseline model* (BL) we compare the dual-source model against, only uses this point estimate and therefore has no free parameters. This model reflects the idea the normative accounts discussed in the introduction share that responses to conditional inferences should come from a coherent probability distribution over the elementary propositions in the inference. We use three estimates from the participants to obtain a prediction for each of the four responses to the conditional inferences: The two estimates of the premises (identical to what is used for obtaining the coherence intervals), which are obtained prior to making the conditional inference, plus the estimate of the alternatives to the conditional, *P*(*q*|¬*p*), which is obtained after making the conditional inference.

For estimating the *dual-source model* (DS), we combined the estimate of the baseline model as knowledge-based component of the dual source model [i.e., ξ(*C*, *x*) in Equation 2] with estimates for the form based component [i.e., τ(*x*) in Equation 2]. As estimates of the form-based components we used participants' responses to the deductive conditional inference task (aggregating across the two different conditionals). These two types of information were integrated using the weighting parameter λ, which we treated as a free parameter (constrained to vary between 0 and 1). In sum, we used four estimates from the participants to obtain predictions for each of the four responses to the conditional inferences (i.e., the three estimates used for the baseline model plus the estimate for the corresponding inference from the deductive task) plus one free parameter per participant.

As the dual-source model now necessarily has to provide at least an as good account of the data as the baseline model (although it uses additional data, the free parameter can only increase the goodness of fit), we considered a variant of the baseline model, denoted BL^*^, which also included one free parameter per participant. Specifically, we wanted to acknowledge the fact that the estimate of alternatives to the conditional, *P*(*q*|¬*p*), was obtained after making the conditional inference. It may well be possible that participants show a bias due to memory or reevaluation effects when giving their estimates of *P*(*q*|¬*p*). Hence, for BL^*^ we estimated one free parameter per participants that was multiplied with all four estimates of *P*(*q*|¬*p*) for that participant and could range between 0 and infinity, therefore acting as a scaling parameter for all four *P*(*q*|¬*p*).

We fitted all three models (BL, DS, and BL^*^) to the data of individual participants (i.e., to the four responses given to the four conditional inferences) using the estimates and parameters described above and using root mean squared deviation (RMSD) of predicted and observed values as criterion. For four data points from four different participants we could not obtain a prediction from the baseline model as a denominator in the formulas given in Footnote 4 was 0. We excluded these four participants from the following analysis.

#### Modeling results

**Figure 5:**
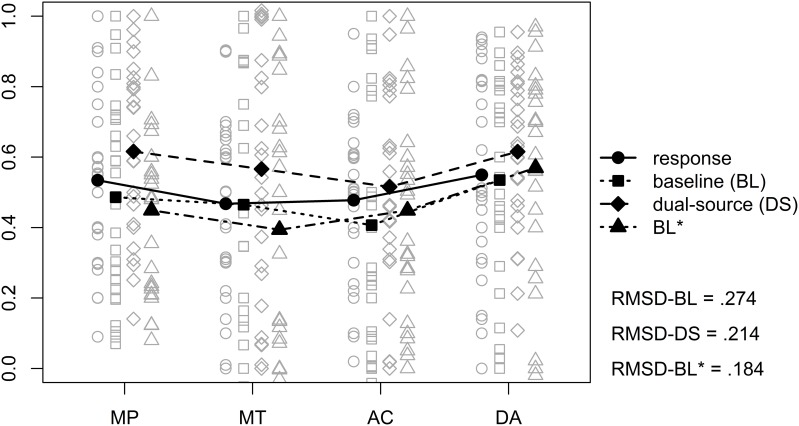
**Mean (filled symbols) and individual (non–filled symbols) model predictions and observed responses from the probabilized conditional inference task**. The solid line shows the observed responses and the different dashed lines show the different models. A small amount of vertical jitter was added to individual data points in case of perfect overlap to make points distinguishable. The values in the lower right corner are the mean root mean squared deviations (RMSD) of the different models vs. the observed responses.

The results from the different models as well as the original responses are displayed in Figure 5, the corresponding mean RMSDs are given in the lower right of the figure. To analyze the results we estimated a LMM on the individual RMSDs with model (baseline, dual-source, and BL^*^) as fixed effect and random intercepts for participants (random slopes for model could not be estimated as our design contained no replicates, Barr, 2013). As expected, we found a significant effect of model, *F*_(2, 50)_ = 9.79, *p* < 0.001. *Post-hoc* tests using Bonferroni-Holm correction for multiple comparisons revealed that, trivially, the models with a free parameter provided a better account than the baseline model, *z* = −4.17, *p* = 0.003. However, there were no differences between the dual-source model and the BL^*^ model, *z* = 1.48, *p* = 0.14.

According to the dual-source model, individuals integrate different types of information when making a conditional inference. An analysis of the estimated λ parameters showed that 81% of the participants used the form-based information (i.e., λ > 0), which ranged for those participants from 0.02 to 0.70 with a mean of 0.31.

An analysis of the free parameter of the BL^*^ model (i.e., the scaling parameter for all *P*(*q*|¬*p*) per participant) indicated that approximately half of the participants (54%) produced too large estimates of *P*(*q*|¬*p*), as indicated by scaling parameters below 1. The median scaling parameter was 0.95 (mean = 1.13, *sd* = 1.18). For three individuals the scaling parameter was even virtually 0, indicating that they did not consider *P*(*q*|¬*p*) at all in their responses to the conditional inferences. The maximal value of the scaling parameter was 6.00.

## General discussion

The goals of this manuscript were to test several central assumptions of what has been introduced as the “new paradigm psychology of reasoning” (Over, 2009). The first question was how individuals understand the conditional. Specifically, we provided another test of the conditional probability hypothesis, addressing the question what predicts the probability of a conditional “If *p* then *q*,” avoiding some limitations of previous assessments. Our results could not be clearer. The data supports the conditional probability hypothesis but none of the alternative explanations. Only *P*(*q*|*p*) adds unique variance to the prediction of *P*(if *p* then *q*). Interestingly, another hypothesis that is associated with the new paradigm but can also be related to causal Bayes nets (e.g., Fernbach and Erb, 2013; Rottman and Hastie, 2014), the delta-p rule, receives essentially no empirical support. This is especially surprising as Douven and Verbrugge (2012) found an effect of a measure similar to delta-p when participants were asked to estimate the acceptability instead of the probability of a conditional. Furthermore, our results extend findings that there is hardly any support for the hypothesis that the conjunction *P*(*p* ∧ *q*) predicts the probability of everyday conditionals (e.g., Over et al., 2007; Douven and Verbrugge, 2013). It seems that this latter hypothesis can only be confirmed for basic conditionals and if participants are not used to the task (Fugard et al., 2011). All in all this shows that for probabilistic tasks as employed here, the Equation offers the only supported explanation as to how participants understand a conditional. If and how causal considerations might also influence this understanding still needs to be shown.

The second main goal was to assess whether two normative accounts that have received special attention within the new paradigm, Adams' (1998) notion of p-validity and Pfeifer and Kleiter's (2005; 2010) mental probability logic, are empirically adequate computational level theories (Marr, 1982) of reasoning. Specifically, we were interested in whether or not individuals' responses are consistent with the norms proposed by the two accounts. Unfortunately, not all of these results can be used as evidence in favor of these accounts. For p-validity it seems that most of the relevant responses (i.e., responses to MP and MT inferences, as p-validity does not restrict responses to AC and DA) are in fact given in accordance to the norm, for MP all responses were even norm conforming. However, when taking the probability into account that responses could be p-valid by chance by considering the smallest response value that would still be p-valid, the analysis shows that only for MP there is above chance performance. For MT, in contrast, performance was at chance level. Similar results were obtained for the intervals predicted by mental probability logic. When taking the size of the interval as chance level into account, only for MP and, to a lesser degree, for DA did participants responses follow the norm. In contrast, for MT and AC only chance performance was observed. Therefore taken together, only the results of MP and of DA for the coherence based approach can be viewed as evidence for the empirical adequacy of p-validity and mental probability logic.

The probabilized conditional reasoning task, albeit allowing us to run a simultaneous by-subject and by-item analysis on directly obtained estimates of all relevant probabilities, contains features which may have undesirable consequences. For example, the eight questions for each conditional are administered in one block, which may have led to anchoring or carry-over effects. Additionally, the questions for *P*(If *p* then *q*), *P*(minor premise), and *P*(conclusion) were always administered in this order and all the other probabilities afterward which may have exacerbated the above problem or induced order effects (this was one reason for the free parameters in the BL^*^ model). Future research could try to rule our these concerns by for example alternating the order or distributing the items per conditional across the experimental session. Note that the sequence of items in the present experiment was in part necessitated by the requirement to present the probability estimates of the premises in the item asking for the probability of the conclusion. Further, it was the sequence least likely to cause undesirable transfer effects in the probabilized conditional inferences which were of central interest here.

Some new paradigm researchers have argued that, by taking a Bayesian approach in the psychology of reasoning, we will find quite a high level of rationality in people, as judged by Bayesian standards (Oaksford and Chater, 2007, 2009). However, other supporters of the new paradigm are doubtful that the new approach will find a very high degree of rationality in people (Evans and Over, 1996, 2013, and see Elqayam and Evans, 2011). Studies in judgment and decision making have found numerous fallacies and biases in people's probability, and also utility, judgments. In our view, it is excessively optimistic to expect these irrational tendencies to disappear completely when people are using their probability judgments, as they commonly do, in their reasoning. From a dual process perspective, one could predict that there will be an increased tendency for higher level processes to be employed in explicit inferences. These higher processes could increase conformity to normative rules, but do not always, or necessarily, do so, whether the rules are probabilistic or not (Elqayam and Over, 2012; Evans and Stanovich, 2013). We would predict some increase in this conformity, but only expect people to be modestly in line with p-validity and coherence in their reasoning (an expectation also confirmed by Evans et al., 2014).

What we found in our experiment is that people were above chance performance only for the MP inference. This finding needs careful assessment and further study. It appears that people are indeed limited to some extent in how far they conform to Bayesian standards. However, we would point out that MP occupies an absolutely central place in Bayesian inference. Take the classic example of Bayesian inference in a scientific procedure. We infer using Bayes' theorem that there is a conditional probability that a certain hypothesis *h* holds given evidence *e*. Recent research, cited above, has shown that people judge the probability of a conditional, *P*(if *e* then *h*), to be the conditional probability, *P*(*h*|*e*). Now the final stage of Bayesian inference is for *e* to be found true or at least probable to some reasonable degree, so that *P*(*e*) is high enough for some confidence in *h*, *P*(*h*), to be inferred. The inference at this last step is usually called conditionalization when *P*(*h*|*e*) is the major premise. We can see that it is an instance of MP when the major premise is the conditional with a degree of belief, *P*(if *e* then *h*) = *P*(*h*|*e*). Bayesian confirmation and belief updating, or belief revision, depend on uses of MP of this general form, when *P*(if *e* then *h*) = *P*(*h*|*e*) is invariant, or rigid, and *P*(*e*) is found to be high (see Chater and Oaksford, 2009; Oaksford and Chater, 2013). For this reason, it is significant that we have found MP performance to be above chance level.

### Conflict of interest statement

The authors declare that the research was conducted in the absence of any commercial or financial relationships that could be construed as a potential conflict of interest.

## Footnotes

^1^Note that Lewis' famous *triviality arguments* (Lewis, 1976) which apparently show that the Equation is untenable on theoretical grounds, actually depend on an interpretation of conditionals that is empirically not supported. See Douven and Verbrugge (2013) for an extensive discussion and experimental results conclusively showing that Lewis' arguments do not apply.

^2^Throughout this manuscript whenever estimating random slopes we also estimated the correlation between the slopes.

^3^An alternative analysis comparing the observed rate of p-valid responses and the chance rate of p-valid responses using either a paired *t* test or a paired permutation test (i.e., stratified by participant) based on 100,000 Monte Carlo samples as implemented in package coin (Hothorn et al., 2006, 2008) yielded the same pattern of significant and non–significant results. However, as this analysis did not take potential effects of the conditionals into account (see e.g., Judd et al., 2012) we prefer to report the LMM analysis.

^4^For MP we used Equation 1. For the other inferences we used the following formulas:

$$\text{MT: }P(\neg p)=\frac{1-P(q|p)-P(\neg q)}{P(q|\neg p)-P(q|p)}\;$$

$$\text{AC: }P(p)=\frac{P(q)-P(q|\neg p)}{P(q|p)-P(q|\neg p)}$$

DA: P(¬q)=1−P(¬p)×P(q|¬p)−P(q|p)(1−P(¬p))

Values outside the probability scale (i.e., outside the interval from 0 to 1) were set to the corresponding border.
