# Supplementary material for: New normative standards of conditional reasoning and the dual-source model
Source: Front Psychol. 2014 Apr 17;5:316. doi: 10.3389/fpsyg.2014.00316 (PMC4029011; doi:10.3389/fpsyg.2014.00316)
Supplement: Supplementary file 1 [file Presentation1.PDF]

Appendix 1

Conditionals used in the reported experiment

No	Conditionals (English translation and German original)
1	If oil prices continue to rise then German petrol prices will rise.<br>Wenn der Ölpreis weiter steigt, dann wird der Sprit in Deutschland teurer werden.
2	If car ownership increases then traffic congestion will get worse.<br>Wenn die Anzahl der Autobesitzer steigt, dann nimmt die Anzahl der Staus zu.
3	If more people use protective sun cream then cases of skin cancer will be reduced.<br>Wenn mehr Menschen Sonnencreme benutzen, dann gibt es weniger Fälle von Hautkrebs.
4	If kindergarten teachers' salaries are improved the recruitment of kindergarten teachers will increase.<br>Wenn die Löhne in Kindergärten steigen, dann werden mehr Erzieher und Erzieherinnen ausgebildet werden.
5	If jungle deforestation continues then Gorillas will become extinct.<br>Wenn die Abholzung der Regenwälder fortschreitet, dann werden Gorillas aussterben.
6	If student grants are raised then university entries will increase.<br>Wenn der BAföG Satz erhöht wird, wird es mehr Studienbewerber geben.
7	If the industrialized nations reduce their CO2 emissions then global warming will be reduced.<br>Wenn die Industrienationen den Ausstoß von CO2 reduzieren, dann wird die globale Erwärmung abgeschwächt.
8	If student fees are brought back then the number of students will drop.<br>Wenn Studiengebühren wieder eingeführt werden, dann wird die Anzahl der Studierenden sinken.
9	If primary school class sizes are reduced then the national education level will improve.<br>Wenn die Klassengröße in der Grundschule reduziert wird, dann wird das allgemeine Bildungsniveau ansteigen.
10	If immigration laws are made stricter then the number of immigrants in Germany will decrease.<br>Wenn die Asylgesetze verschärft werden, dann wird die Anzahl der Imigranten in Deutschland abnehmen
11	If the cost of fruit and vegetables is subsidised then people will eat more healthily.<br>Wenn die Preise für Obst und Gemüse subventioniert werden, dann werden die Menschen sich gesünder ernähren
12	If German troops remain in Afghanistan then acts of terrorism in Germany will increase.<br>Wenn deutsche Soldaten in Afghanistan bleiben, dann wird es in Deutschland mehr terroristische Anschläge geben.
13	If genetic research continues then a cure for any cancer will be found.<br>Wenn die Genforschung fortgesetzt wird, dann wird es eine Behandlung für jeden Krebs geben.
14	If the cost of fuel increases then more people in Freiburg will use bicycles.<br>Wenn die Spritpreise weiter steigen, dann werden mehr Menschen in Freiburg Fahrrad fahren.
15	If global warming continues then Hamburg will be flooded.<br>Wenn die globale Erwärmung weiter anhält, dann wird Hamburg überschwemmt werden.
16	If Greece leaves the Euro then Italy will too.<br>Wenn Griechenland den Euro verlässt, dann wird Italien den Euro verlassen.

Note. Conditionals 1 to 13 are adapted from Evans et al. (2010).

Appendix 2

Linear Mixed Model with Interactions on the Probability of the Conditional $P(\text{if } p \text{ then } q)$

Effect	Parameter	F	*df*	F-scaling	p
(Intercept)	0.01	0.08	1, 22.75	1	.78
Inference		0.22	3, 10.10	0.84	.88
$P(q\|p)$	0.69	35.15	1, 84.82	1	<.001
$P(p \wedge q)$	0.04	0.14	1, 88.76	1	.71
$P(\neg p \vee q)$	0.18	2.35	1, 81.39	1	.13
$P(q\|\neg p)$	-0.20	2.51	1, 85.92	1	.12
$P(q\|p) \times P(p \wedge q)$	-0.20	0.76	1, 75.03	1	.39
$P(q\|p) \times P(\neg p \vee q)$	0.31	0.43	1, 74.33	1	.51
$P(p \wedge q) \times P(\neg p \vee q)$	-0.74	2.89	1, 73.03	1	.09
$P(q\|p) \times P(q\|\neg p)$	-0.30	0.58	1, 83.40	1	.45
$P(p \wedge q) \times P(q\|\neg p)$	0.38	0.93	1, 79.76	1	.34
$P(\neg p \vee q) \times P(q\|\neg p)$	0.67	3.26	1, 83.72	1	.07
$P(q\|p) \times P(p \wedge q) \times P(\neg p \vee q)$	-1.98	4.09	1, 72.74	1	.047
$P(q\|p) \times P(p \wedge q) \times P(q\|\neg p)$	1.13	1.26	1, 85.01	1	.27
$P(q\|p) \times P(\neg p \vee q) \times P(q\|\neg p)$	-0.67	0.20	1, 73.96	1	.66
$P(p \wedge q) \times P(\neg p \vee q) \times P(q\|\neg p)$	0.73	0.28	1, 75.76	1	.60
$P(q\|p) \times P(p \wedge q) \times P(\neg p \vee q) \times P(q\|\neg p)$	-4.02	2.02	1, 71.08	1	.16

Note. The model was fitted with restricted maximum likelihood. Model *df* = 31, AIC = -91.91, BIC = -5.50, deviance = -153.91, Ω_0^2 = .87 (explained variance against the intercept only model; Xu, 2003).
